# Nucleotide metabolism-related host proteins RNA polymerase II subunit and uridine phosphorylase 1 interacting with porcine epidemic diarrhea virus N proteins affect viral replication

**DOI:** 10.3389/fvets.2024.1417348

**Published:** 2024-06-12

**Authors:** Yifan Xu, Heyou Yi, Qiyuan Kuang, Xiaoyu Zheng, Dan Xu, Lang Gong, Liangyu Yang, Bin Xiang

**Affiliations:** ^1^College of Veterinary Medicine, Yunnan Agricultural University, Kunming, China; ^2^College of Veterinary Medicine, South China Agricultural University, Guangzhou, China; ^3^Key Laboratory of Animal Pathogen Infection and Immunology of Fujian Province, College of Animal Sciences, Fujian Agriculture and Forestry University, Fuzhou, China

**Keywords:** porcine epidemic diarrhea virus, N protein, RPB2, UPP1, protein interaction

## Abstract

Porcine epidemic diarrhea virus (PEDV) is a highly infectious pathogen that targets pig intestines to cause disease. It is globally widespread and causes huge economic losses to the pig industry. PEDV N protein is the protein that constitutes the core of PEDV virus particles, and most of it is expressed in the cytoplasm, and a small part can also be expressed in the nucleus. However, the role of related proteins in host nucleotide metabolic pathways in regulating PEDV replication have not been fully elucidated. In this study, PEDV-N-labeled antibodies were co-immunoprecipitated and combined with LC-MS to screen for host proteins that interact with N proteins. Bioinformatics analyses showed that the selected host proteins were mainly enriched in metabolic pathways. Moreover, co-immunoprecipitation and confocal microscopy confirmed that the second-largest subunit of RNA polymerase II (RPB2) and uridine phosphorylase 1 (UPP1) interacted with the N protein. RPB2 is the main subunit of RNA polymerase II and plays an important role in eukaryotic transcription. UPP1 is an enzyme that catalyzes reversible phosphorylation of uridine to uracil and ribo-1-phosphate to promote catabolism and bio anabolism. RPB2 overexpression significantly promoted viral replication, whereas UPP1 overexpression significantly inhibited viral replication. Studies on interactions between the PEDV N and host proteins are helpful in elucidating the pathogenesis and immune escape mechanism of PEDV.

## 1 Introduction

Porcine epidemic diarrhea is an acute, highly contagious disease of pigs caused by the porcine epidemic diarrhea virus (PEDV). Newborn piglets infected with PEDV exhibit diarrhea, dehydration, vomiting, and high mortality (1). PEDV was first reported in the United Kingdom in 1971 and has since become globally widespread in the pig industry. After a large-scale outbreak of PEDV variant strains in China in 2010, huge economic losses were incurred by the pig industry (2–5).

PEDV belongs to the coronavirus family and is a plus-stranded RNA virus with a total genome length of approximately 28 kb. It encodes 16 nonstructural and four structural proteins: spike (S protein), membrane (M protein), envelope (E protein), and nuclear (N protein) proteins, as well as one helper protein (ORF3) (6). The structural N protein is the core protein of the virion, which wraps around the RNA genome of the virus and forms a helical ribonucleoprotein with an RNA chaperone activity (7, 8). The N protein is localized in microparticles throughout the cytoplasm of coronavirus-infected cells and can also be localized in the nucleolus of some cells (9). N proteins may also stabilize the envelope assembly complex during VLP assembly by interacting with M proteins (10). Studies have shown that the coronavirus N protein can regulate host protein expression. The SARS-CoV N protein can up-regulate the host COX2 protein, causing inflammation through multiple COX-2 signaling cascades (11, 12). The PEDV N protein interacts with the host autophagy pathway to degrade HNRNPA1, FUBP3, HNRNPK, PTBP1, and TARDBP proteins, thereby promoting PEDV replication (13). The PEDV N protein can degrade STAT1 and prevent its phosphorylation, thus inhibiting interferon-stimulated gene expression, which is conducive to self-replication (14). The PEDV N protein interacts with host p53 protein to induce S-phase arrest, thereby promoting viral replication (15). The PEDV N protein promotes the cyclization of viral mRNA carried by the N protein through interactions with PABPC1 and eIF4F proteins, thus promoting viral transcription and replication (13, 16). However, the role of related proteins in host nucleotide metabolic pathways in regulating PEDV replication is still unknown. RNA polymerase II largest subunit (RPB2) and uridine phosphorylase 1 (UPP1) are key proteins in the nucleotide metabolic pathway. RPB2 regulates the activity of RNA polymerase (17) and UPP1 regulates the activity of thymidine phosphorylase (18). In this study, co-immunoprecipitation (Co-IP) and LC-MS were used to screen and identify host protein profiles that interact with PEDV-N. It was found that PEDV N protein interacts with host proteins RPB2 and UPP1, which are related to nucleotide metabolism, aiming to supplement the function of the PEDV N protein, and to further understand the infection mechanism of PEDV to provide a scientific basis for the development of PED prevention and control strategies.

## 2 Materials and methods

### 2.1 Cells, viruses, and plasmids

Vero-E6 cells and HEK293T cells were cultured in DMEM (Gibco, Guangzhou, China) with 10% serum (Gibco, Guangzhou, China) at 37°C and 5% CO_2_. The PEDV used in this study was the newly isolated and identified FS202201 strain (19), which was maintained in infected cells in DMEM containing 7 μg/mL trypsin (Gibco, Guangzhou, China).

The Fastagen kit (Fastagen, Shanghai, China) was used to extract whole genome RNA from PEDV and Vero-E6 cells, according to the manufacturer’s instructions, and GenStar reverse transcriptase (Genstar, Guangzhou, China) used to reverse-transcribe the RNA into cDNA, which was used as a template to amplify the target gene fragment via PCR. The primers used are listed in Table 1. PCAGGS-N-HA and PCAGGS-RPB2/UPP1-Flag plasmids were constructed using the recombinant enzyme (C112) (Vazyme, China, Shanghai) from the target gene and pCAGGS vector cut by the enzyme. All plasmids were verified using sequencing.

**Table 1:** Primer sequences used to construct plasmids.

Primers	Sequences (5′–3′)
pCAGGS-N-HA-F	ATGGCTTCTGTCAGCTTCCA
pCAGGS-N-HA-R	AATTAAAGGACATAGCTTCTA
pCAGGS-RPB2-Flag-F	ATGTCCACTCCCCCAGCCACCG
pCAGGS-RPB2-Flag-R	AATGTGAGAGTGCGAGTGCGGTCTT
pCAGGS-UPP1-Flag-F	AGACTCCCTATGAGCTTCACCT
pCAGGS-UPP1-Flag-R	AAAACCTGTCACGAAAATTA

### 2.2 Reagents and antibodies

Lipofectamine 2000 (11668500) was purchased from Thermo Fisher Scientific (Shanghai, China). GAPDH, Flag, and the mouse anti-HA monoclonal antibodies (M20003) were purchased from Abmart (Shanghai, China), and CoraLite 594-conjugated goat anti-mouse IgG (H + L) and CoraLite 488-conjugated goat anti-rabbit IgG (H + L) antibodies obtained from Proteintech (Proteintech, Guangzhou, China). Anti-PEDV N protein mouse monoclonal antibody was prepared in our laboratory. The endonuclease sites used for plasmid construction are ECoRI (Thermo, FD0274) and SacI (Thermo, FD1134).

### 2.3 Immunoprecipitation

Vero-E6 cells were inoculated into a 10 cm cell culture dish and transfected with the pCAGGS-N-HA plasmid using Lipofectamine 2000. Proteins were extracted 24 h later using RIPA lysis buffer (P0013B Biotronix) containing a protease phosphatase inhibitor mixture (P1048 Biotronix). The proteins were incubated at 4°C for 15 min, centrifuged at 15,000 × g for 10 min, and the supernatant thereafter removed. The cell lysate was added to HA-labeled magnetic beads that were washed with TBS and thereafter incubated in a 4°C shaker for 12 h. The samples were then subjected to mass spectrometry (MS) analysis.

### 2.4 LC-MS analysis

The magnetic beads were centrifuged and the supernatant discarded. The magnetic beads were washed twice with 200 μL 1× PBS. A 100 μL volume of a 50 mmol/L NH_4_HCO_3_ solution was added to resuspend the magnetic beads. The final concentration of DTT solution was 10 mmol/L, and the solution reduced in a water bath at 56°C for 1 h. The final concentration of the IAA solution was 50 mmol/L, and the reaction incubated in the dark for 40 min. Trypsin was added according to the mass ratio of trypsin to substrate (1:100), and the enzyme added at 37°C for 4 h. The enzyme was further added according to the mass ratio (1:100), and the enzyme digestion reaction left overnight at 37°C for 16 h. After digestion, the peptides were desalted using a self-filling column, and the solvent dried in a vacuum centrifuge concentrator at 45°C. The peptide was dissolved with the sample solution (0.1% formic acid, 2% acetonitrile), then fully oscillated in a vortex, centrifuged at 13,200 rpm for 10 min at 4°C, and the supernatant thereafter transferred to the upper sample tube for mass spectrometry analysis. The samples were detected by a Q Exactive Hybrid Quadrupole-Orbitrap Mass Spectrometer (Thermo Fisher Scientific), and the relevant parameters are shown in Table 2. The mass spectrometry proteomics data have been deposited to the ProteomeXchange Consortium via the PRlDE (20) partner repository with thedataset identifier PXD052564.

**Table 2:** Parameters used for mass spectrometry analysis.

Mass spectrometry	
Spray voltage	2.2 kV
Capillary temperature	270°C
MS resolution	70,000 at 400 *m*/*z*
MS precursor *m*/*z* range	300.0–1800.0
Product ion scan range	start from m/z 100
Activation type	CID
Min. signal required	1500.0
Isolation width	3.00
Normalized coll. energy	40.0
Default charge state	6
Activation *Q*	0.250
Activation time	30.000
Data dependent MS/MS	Up to top 20 most intense peptide ions from the preview scan in the Orbitrap

### 2.5 Biological information analysis

Raw MS files were analyzed and searched against the target protein database based on the sample species using MaxQuant (1.6.2.10). OmicsBean software^1^ was used to annotate functional classifications of the proteins. KEGG pathway annotations were analyzed using Kobas 3.0.

### 2.6 Co-immunoprecipitation assay

HEK293T cells were inoculated into a 10 cm cell culture dish and co-transfected with the pCAGGS-N-HA and targeted host gene expression plasmids (pCAGGS-RPB2-Flag, pCAGGS-UPP1-Flag) using Lipofectamine 2000. Proteins were extracted 24 h later, as described in section 2.3. The proteins were incubated at 4°C for 15 min, then centrifuged at 15,000 × g for 10 min, whereafter the supernatant was removed. The cell lysate was added to HA-labeled magnetic beads that were washed with TBS and incubated in a 4°C shaker for 12 h. The beads were washed four times with cold PBST, and 1× SDS loading buffer diluted with cell lysate was added and heated in a metal bath at 100°C for 5 min, whereafter SDS-PAGE was performed.

### 2.7 Transfection

Vero-E6 cells and HEK293T cells were seeded onto 12-well plates and transfected when they reached 80% confluency. A 100 μL volume of serum-free Opti-MEM medium and 2 μg plasmid were added into a 1.5 mL EP tube and gently mixed. In another 1.5 mL EP tube, 200 μL serum-free Opti-MEM medium and 6 μL Lipofectamine 2000 transfection reagent were added and gently mixed. After incubation at 25°C for 5 min, the contents of both tubes were gently mixed and incubated at 25°C for 15 min. The culture medium was discarded, the cells gently washed with PBS once, and the incubated mixture added and incubated in a cell incubator at 37°C with 5% CO_2_ for 6 h. Thereafter, the culture medium was replaced with 1 mL DMEM containing 2% FBS (Gibco) and incubated in a cell incubator at 37°C with 5% CO_2_ for 24 h.

### 2.8 Immunofluorescence assay

When Vero-E6 cells reached 80% confluency, they were co-transfected with the pCAGGS-N-HA and targeted host gene expression plasmids (pCAGGS-RPB2-Flag, pCAGGS-UPP1-Flag) for 24 h, whereafter they were fixed with 4% paraformaldehyde at 25°C for 15 min and permeated with 0.2% Triton X-100 at 25°C for another 15 min. The cells were incubated with specific antibodies at 4°C overnight or at 37°C for 1 h. Thereafter, they were incubated with CoraLite 488-conjugated goat anti-mouse and CoraLite 594-conjugated goat anti-rabbit secondary antibodies diluted with PBS at 37°C for 45 min, and their nuclei stained with DAPI for 5 min at 25°C. The cells were cleaned with PBS three times before each operation. Cells were observed under a fluorescence microscope (Leica, Wetzlar, Germany).

### 2.9 Western blotting

Proteins were separated on a 10% SDS-PAGE gel (Vazyme, Shanghai, China) and transferred to polyvinylidene fluoride membranes. We used 5% skim milk powder enclosed in a shaker at 25°C for 1 h to prevent nonspecific binding. The specific PEDV N protein, HA, Flag and GAPDH primary antibodies were incubated at 25°C for 1 h, and thereafter incubated with the corresponding IRDye 800CW secondary antibody at 25°C for 1 h. After closure, samples were washed with TBST buffer three times before each step. The results were observed using a Sapphire RGBNIR Biomolecular Imager (Azure Biosystems, Dublin CA, United States).

### 2.10 Prediction site analysis

The tertiary structure of the PEDV N protein (GenBank: WMT38788.1) was predicted using Alphafold2. The N protein model with the highest accuracy was selected according to the predicted local distance difference test, and HADDOCK 2.4 used to predict the interaction between the two host proteins, RNA polymerase II (RPB2) (GenBank: EHH53784.1) and uridine phosphorylase 1 (UPP1) (GenBank: EHH52134.1). Host protein sequences were obtained from the PDB database. The optimal interaction model was selected based on docking parameters, including the affinity index of the protein-ligand complex, contact residue ratio, and van der Waals force, as well as the electrostatic, confinement, and dissolution energies. The types of polar bonds, accessible and buried surface areas, and folding free energies of potential amino acid interaction sites in the interaction model were predicted using PDBePISA. PyMOL was used to demonstrate the three-dimensional structure of the interaction model, in which the polar bond between the 5A viral and host proteins was selected for amino acid interactions, and the interaction site with the highest confidence obtained according to the PDBePISA results.

### 2.11 Statistical analysis

Data were analyzed using GraphPad Prism 7.0, and all data expressed as the mean ± standard deviation. Student’s *t*-test was used to determine whether differences between the mean values were statistically significant (*p* < 0.05).

## 3 Results

### 3.1 Co-IP-MS analysis of the PEDV N protein

**Figure 1:**
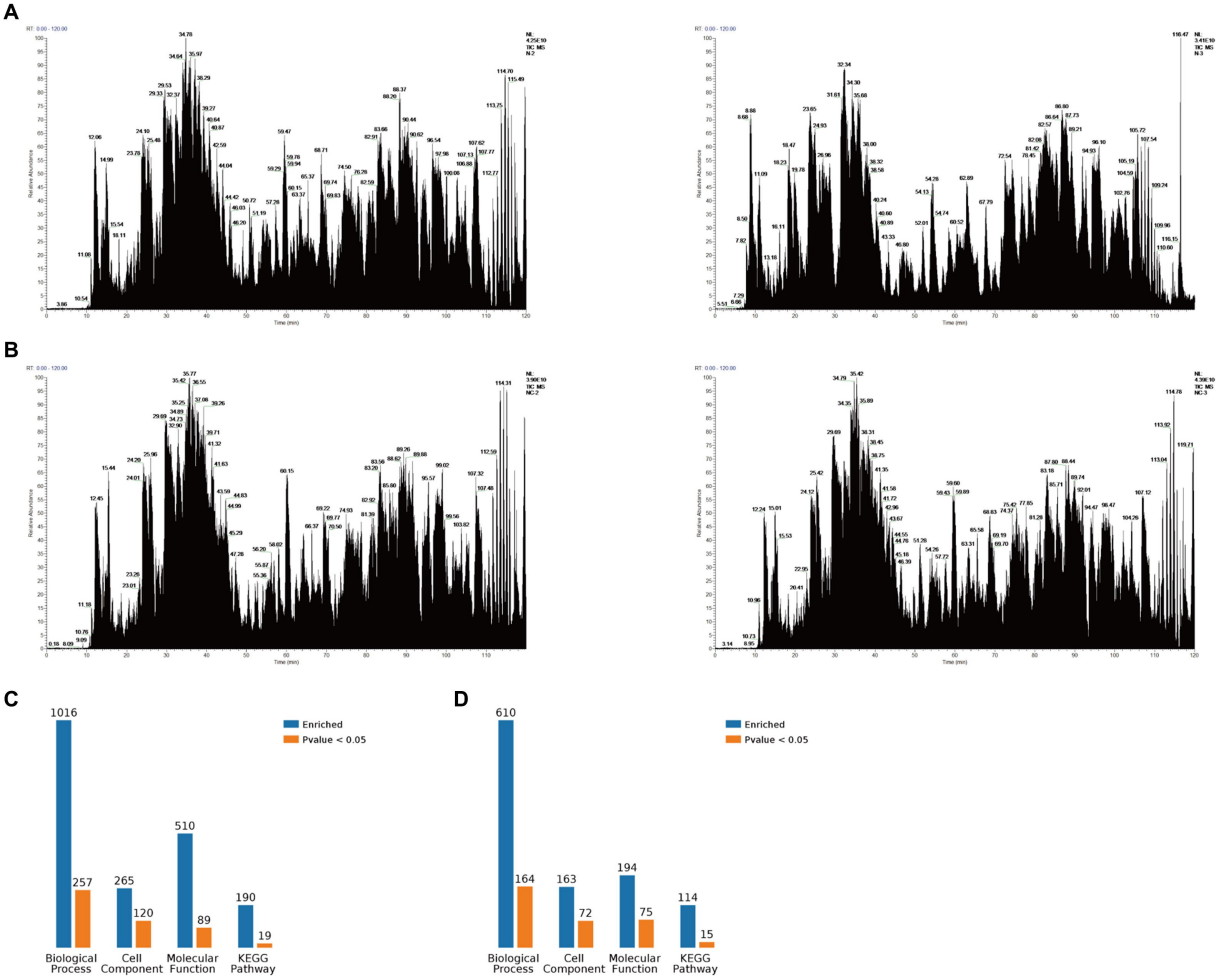
Mass spectrometry data. **(A)** Total ion flow chromatogram obtained via mass spectrometry after Vero-E6 cells were transfected with a PEDV-N protein expression plasmid and co-immunoprecipitation (Co-IP), including two replicates. **(B)** Total ion flow chromatogram obtained via mass spectrometry after Vero-E6 cells were transfected with an empty plasmid and Co-IP, including two replicates. **(C)** Classification of N proteome-related proteins. **(D)** Classification of related proteins compared between the N protein and no-load control groups.

The PEDV N protein expression and no-load plasmids were transfected into Vero-E6 cells, and the PEDV N protein pulled down via Co-IP for Co-IP-MS detection. The treated samples were analyzed using LC-MS; the raw file of the original mass spectrometry results was obtained, and the total ion flow chromatogram (Figures 1A,B) generated after analysis using MaxQuant (1.6.2.10). The total ion flow diagram showed that the number of peaks was large and the peak width small, which indicates that the separation efficiency of liquid chromatography was good, the mass spectrometry data collection normal, and the parallelism good. Compared with the control group, 791 different proteins were enriched, of which 144 were significantly differentially expressed proteins. These differential proteins were enriched by KEGG pathway to 114 pathways, 11 of which were significant differences, including metabolic pathways, biosynthesis of amino acids, pyrimidine metabolism, purine metabolism, metabolism od xenobiotics by cytochrome P450, RNA polymerase, RNA transport, FoxO signaling pathway, Hippo signaling pathway, cell cycle, adipocytokine signaling pathway. At the same time, the differential proteins were subjected to Gene Ontology (GO) functional enrichment analysis based on biological process (BP), cellular component (CC), and molecular function (MF). The results showed significant enrichment in BP related ribonucleoprotein complex assembly, ribonucleoprotein complex subunit organization, macromolecular complex subunit organization, ribonucleoprotein complex biogenesis, cellular macromolecular complex assembly, cellular localization, intracellular transport, cellular component biogenesis, cellular component assembly, protein localization. CC related intracellular part, intracellular, cytoplasm, cell, cytoplasm, macromolecular complex, intracellular organelle, organelle, cytoplasmic part, protein complex. MF related small molecule binding, nucleotide binding, nucleoside phosphate binding, RNA binding, ribonucleoside binding, nucleoside binding, carbohydrate derivative binding, purine ribonucleoside triphosphate binding, purine ribonucleotide binding, purine nucleotide binding (Figures 1C,D). The LC-MS data was derived from previous research (21).

### 3.2 GO functional enrichment analysis of PEDV N-interacting proteins

**Figure 2:**
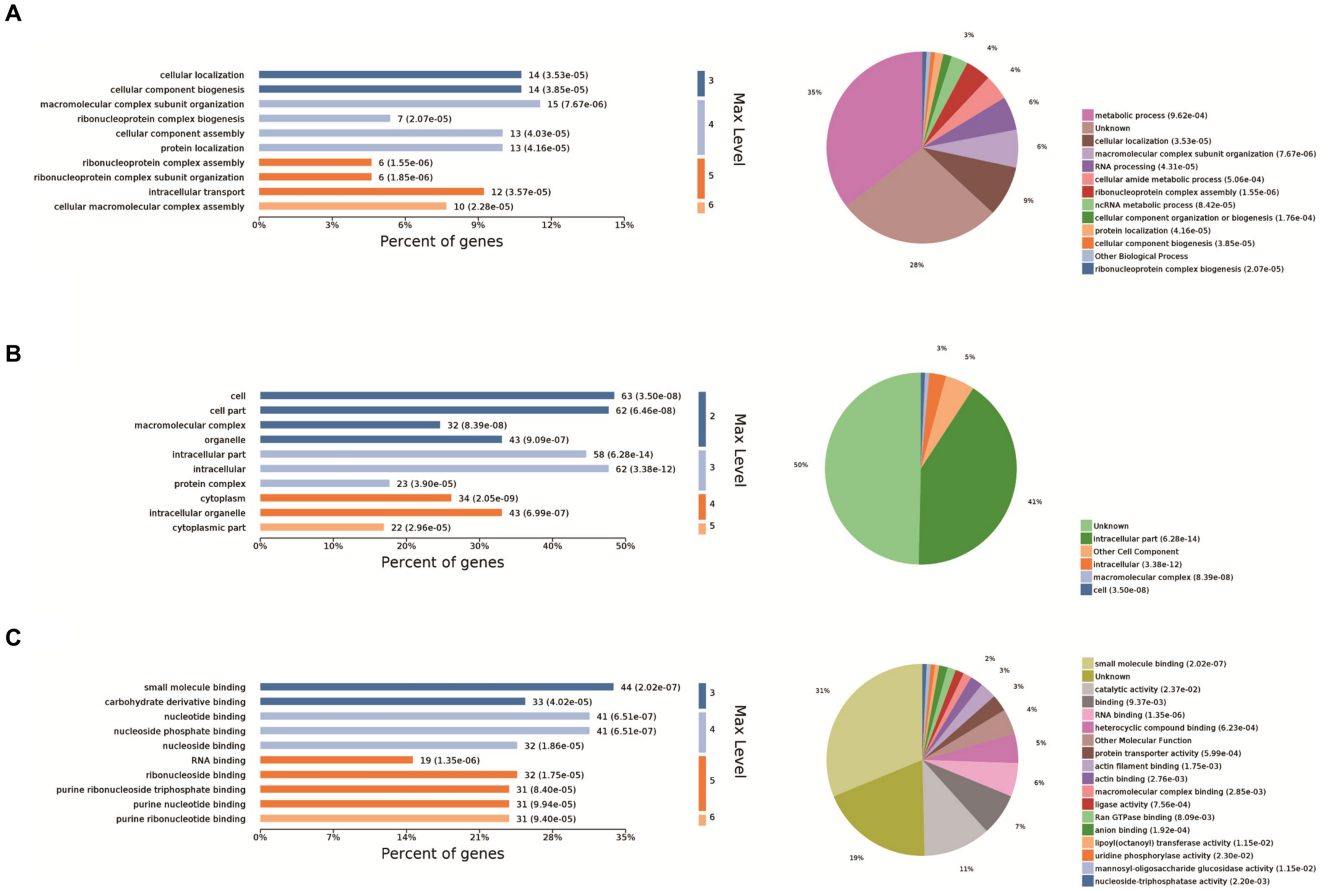
GO analysis. The 10 most significant GO nodes are shown, and the biological processes that each protein is most likely to participate in were counted and represented as pie charts. The horizontal coordinate of the bar chart in the figure is the percentage of enriched proteins, and the number after each bar is the number of proteins in that classification. Pie charts are the biological processes that each protein is most likely to be involved in based on a *p*-value. **(A)** Biological process, **(B)** cellular component, and **(C)** molecular function categories of the proteins.

The 10 most significant GO functions at different maximum levels were selected in the biological process, cellular component, and molecular function categories, and the number and percentage of proteins related to each function represented by bar charts. Based on the *p*-value, the biological process in which each protein was most likely to be involved was determined, and pie charts drawn based on the results to clearly determine the percentage of different proteins in each group. Compared with the control group, there were 610 biological processes, most of which were related to metabolic processes. It also enriched ribonucleoprotein complex assembly, ribonucleoprotein complex subunit organization and macromolecular complex subunit organization, ribonucleoprotein complex biogenesis, cellular macromolecular complex assembly, cellular localization, intracellular transport, cellular component biogenesis, cellular component assembly, protein localization. Biological processes also have the largest percentage of proteins involved in metabolic processes (35%), cellular localization (9%), macromolecular complex subunit organization (6%), RNA processing (6%), cellular amide metabolic process (4%), ribonucleoprotein complex assembly (4%), ncRNA metabolic process (3%) (Figure 2A). The cell components were enriched to 163 related nodes, among which the intracellular part was the most important. In addition, there were significant differences in intracellular organelle, cytoplasm, cell, cytoplasm, macromolecular complex, intracellular organelle, organelle, cytoplasmic part, protein complex. Most of the proteins were associated with the intracellular part (41%), other cell component (5%), and intracellular (3%) (Figure 2B). The molecular function is enriched to 194 nodes, and small molecule binding is the most important node. In addition, there are significant differences in nucleotide binding, nucleoside phosphate binding, RNA binding, ribonucleoside binding and nucleoside binding, carbohydrate derivative binding, purine ribonucleoside triphosphate binding, purine ribonucleotide binding, purine nucleotide binding. Small molecule binding was associated with the most proteins (31%), catalytic activity (11%), binding (7%), RNA binding (6%), heterocyclic compound binding (5%), other molecular function (4%), protein transporter activity (3%), actin filament binding (3%), and actin binding (2%) (Figure 2C).

### 3.3 KEGG pathway enrichment analysis of PEDV N-interacting proteins

**Figure 3:**
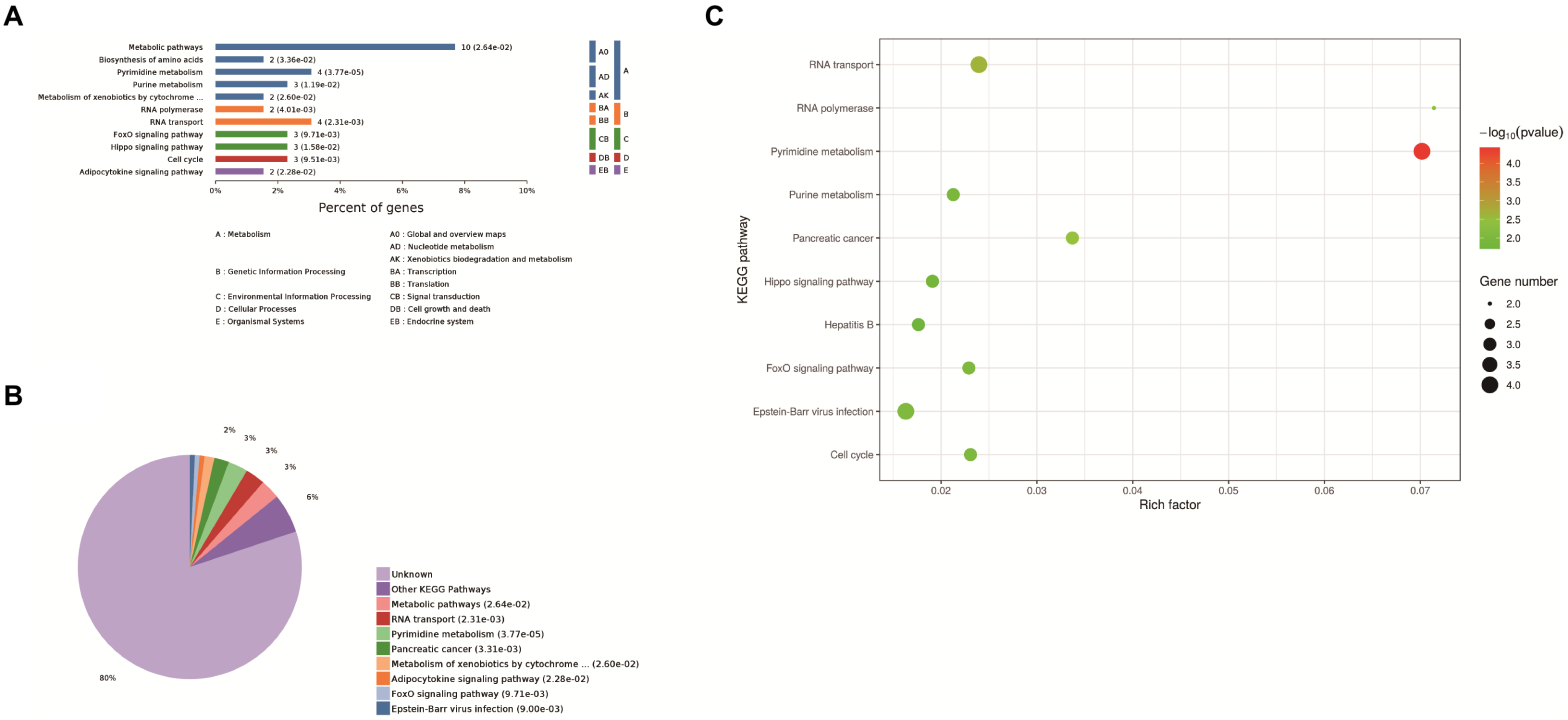
KEGG analysis. **(A)** Enrichment category of the KEGG pathway. The horizontal coordinate is the percentage of enriched protein, and the vertical coordinate is the largest level from smallest to largest. Different levels are shown in different colors, and the number behind each column is the number of proteins in that category. **(B)** Classification and statistics of the KEGG pathway of expressed proteins. **(C)** Bubble map of the KEGG pathway of differentially expressed proteins. Top-10 Kyoto Encyclopedia of Genes and Genomes (KEGG) enriched pathways of differentially expressed genes (DEGs) between Control group and PEDV N protein overexpression group. In the figure, the horizontal coordinate KEGG Term represents the name of the pathway in which the protein is enriched. The ordinate rich factor represents the enrichment factor, and the larger the rich factor, the higher the enrichment degree. Protein number on the right side of the legend indicates the number of proteins enriched by the pathway.

Eleven enrichment classes of the KEGG pathways with the most significant differences were shown. These include Metabolic pathways, biosynthesis of amino acids, pyrimidine metabolism, purine metabolism, and metabolism od xenobiotics by cytochrome P450, RNA polymerase, RNA transport, FoxO signaling pathway, Hippo signaling pathway, cell cycle, adipocytokine signaling pathway (Figure 3A). Based on the *p*-value, we determined the biological process in which each protein was most likely involved. It mainly includes metabolic pathways, RNA transport, pyrimidine metabolism, pancreatic cancer and metabolism od xenobiotics by cytochrome P450, FoxO signaling pathway, Epstein–Barr virus infection, adipocytokine signaling pathway (Figure 3B). We found that host proteins that interact with N proteins are mainly involved in RNA transport, RNA transport, Pyrimidine metabolism, and Purine metabolism. Finally, according to the bubble map, the pyrimidine and urine metabolism pathways (Figure 3C) were selected based on the *p*-value, degree of enrichment, and number of proteins enriched in the pathway.

### 3.4 Protein–protein interaction network analysis of PEDV N-interacting proteins

**Figure 4:**
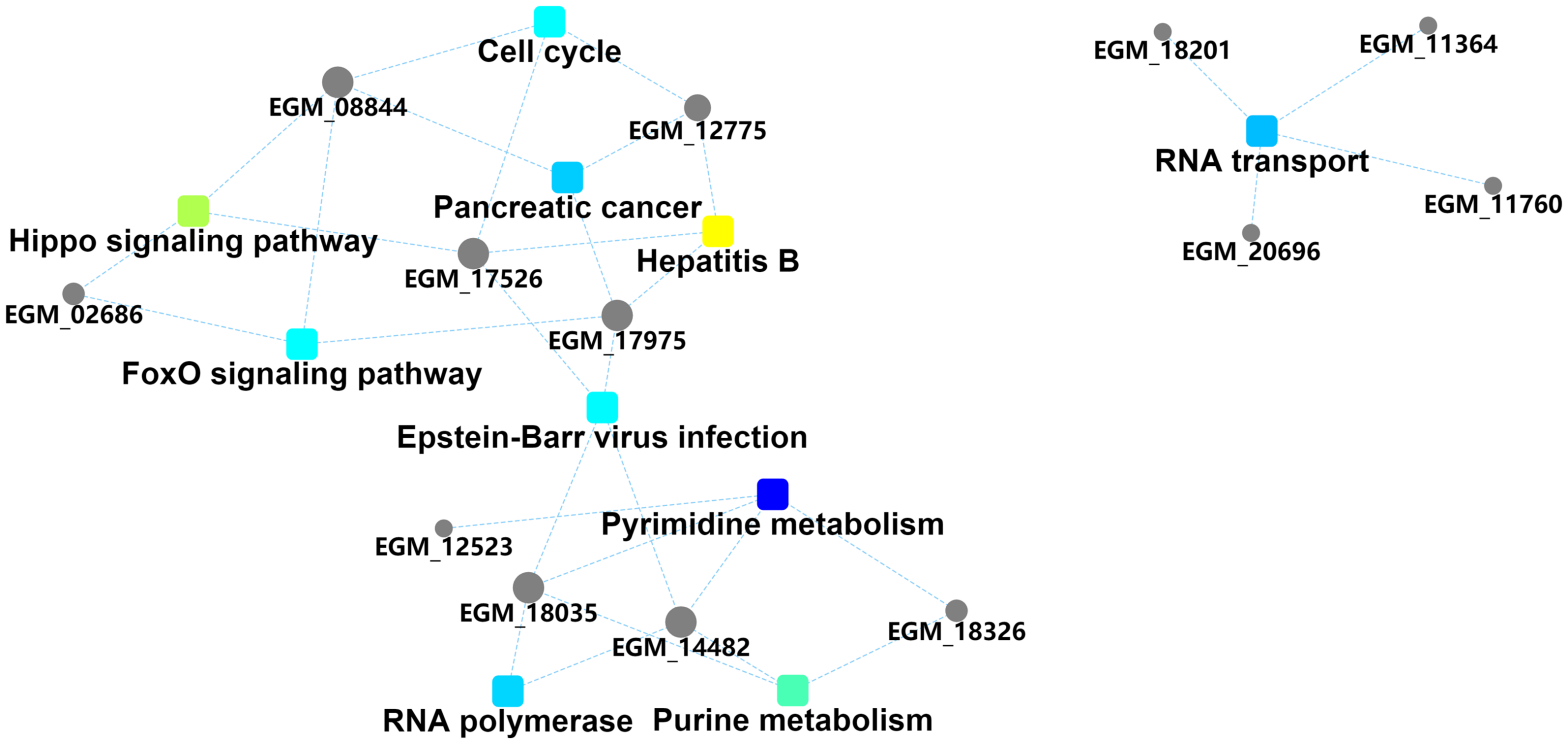
Protein–protein interaction information. Squares represent GO/KEGG terms and circles represent genes/proteins.

The interaction diagram of the differentially expressed proteins demonstrated the importance of pyrimidine and purine metabolism, which could interact with four and three host proteins, respectively, and are thus associated with other biological processes (Figure 4). Pyrimidine and purine metabolism mainly involves the anabolism of pyrimidine and purine nucleotides. These results suggest that the PEDV N protein may create favorable conditions for viral replication and proliferation by regulating host nucleotide metabolic pathways.

### 3.5 Verification of the interaction between PEDV N and two host proteins

**Figure 5:**
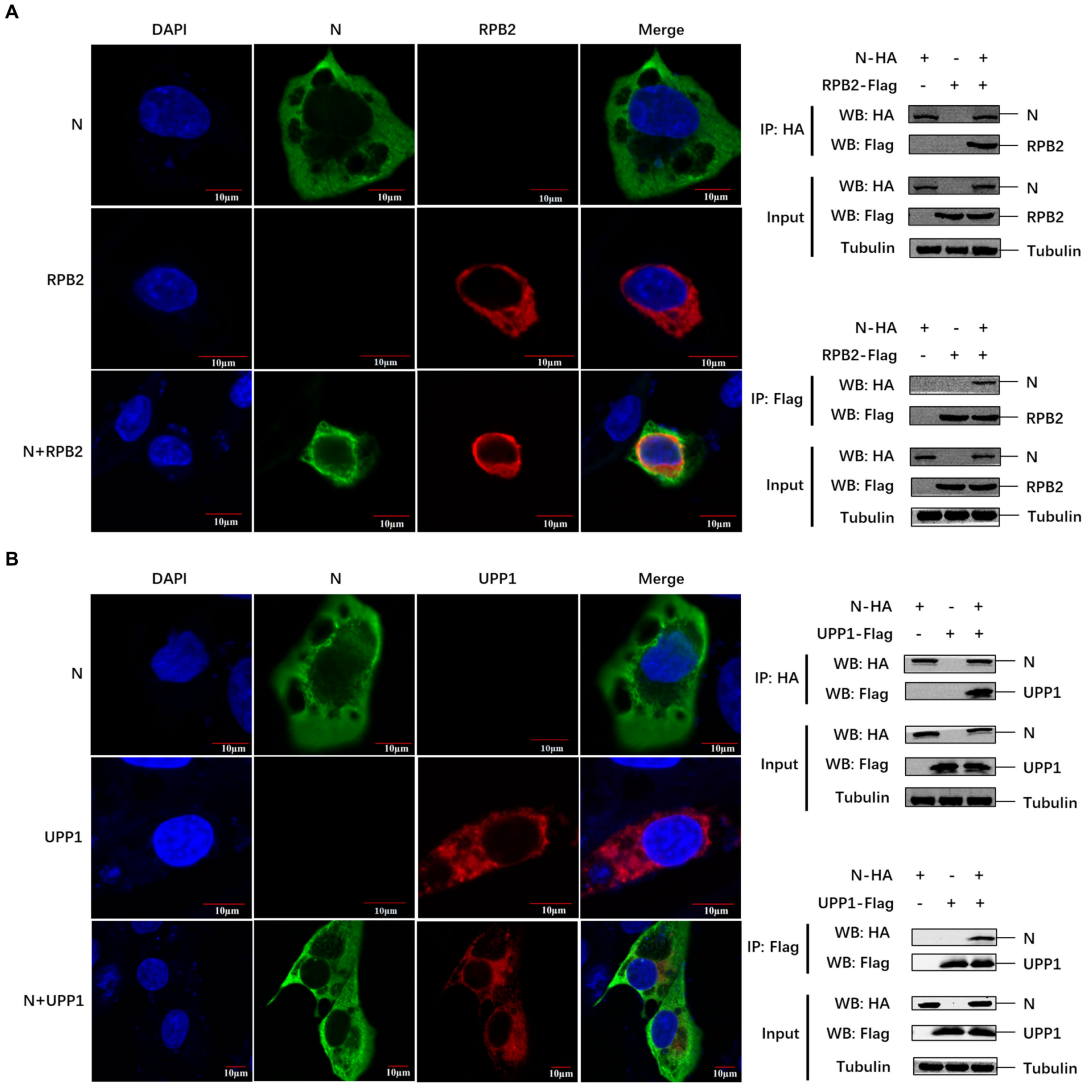
The PEDV N protein interacts with RPB2 and UPP1 host proteins. **(A,B)** The subcellular localization of PEDV N protein and host protein RPB2 and UPP1 in Vero-E6 cells was detected by confocal. The interaction between PEDV N protein and host protein RPB2 and UPP1 was detected by Co-IP.

We further verified the relationship between the PEDV N protein and the two identified pathways. Known proteins in the two pathways (RPB2 and UPP1) were selected to verify their interaction with the PEDV N protein. Confocal microscopy was used to detect the colocalization between PEDV N and host RPB2 and UPP1 proteins, and the results showed that there was a colocalization phenomenon between in Vero-E6 cells, which was further demonstrated via Co-IP in HEK293T cells that PEDV N interacts with RPB2 and UPP1, respectively (Figures 5A,B).

### 3.6 RPB2 and UPP1 participates the regulation of virus replication

**Figure 6:**
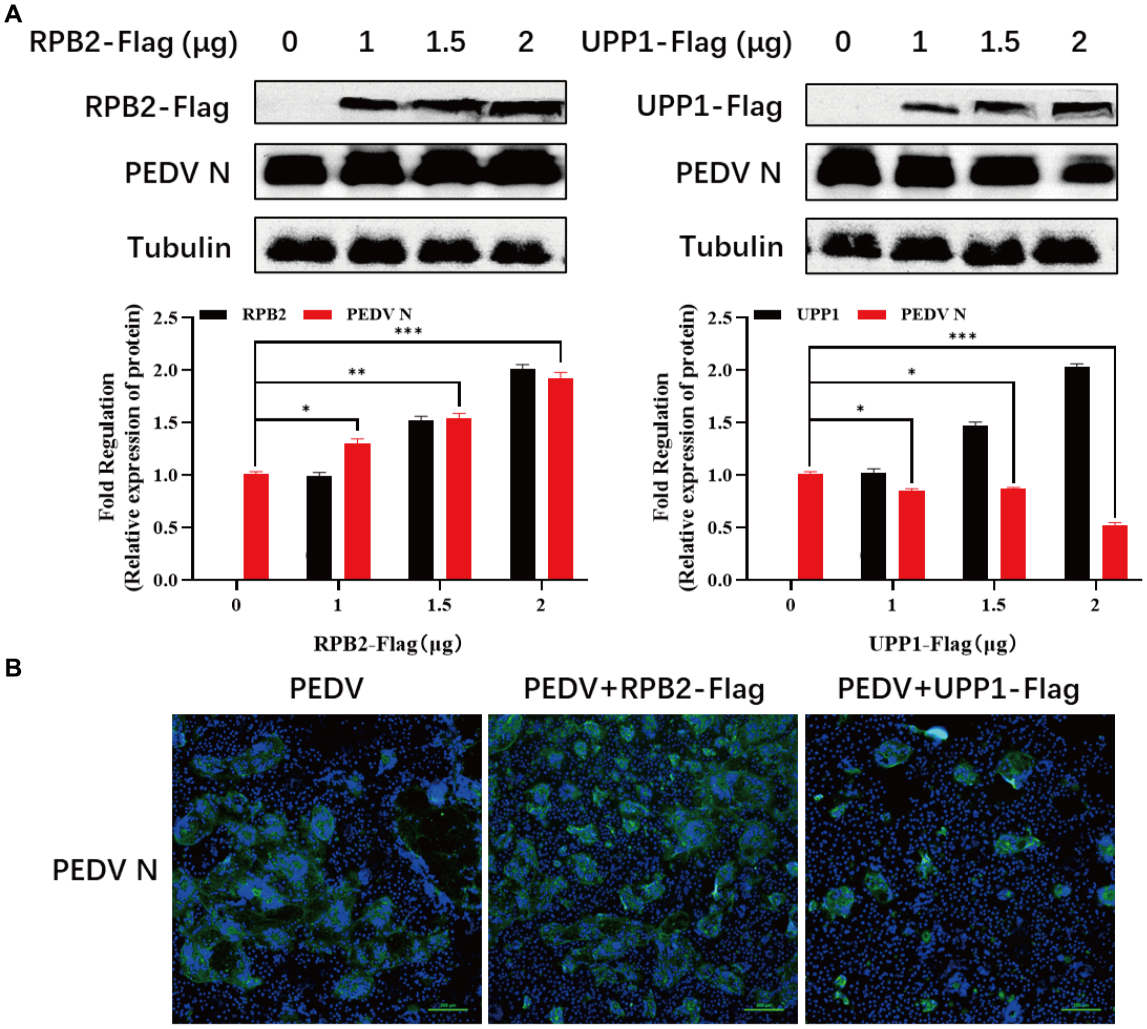
RPB2 and UPP1 overexpression inhibits PEDV replication. **(A)** After overexpression of 1 μg, 1.5 μg, and 2 μg RPB2 and UPP1 in Vero-E6 cells, PEDV was infected and the expression level of PEDV N protein was detected. **(B)** After 2 μg RPB2 and UPP1 was overexpressed in Vero-E6 cells, PEDV was infected and the PEDV-N protein-specific green fluorescence and the syncytia was detected.

The RPB2 and UPP1 plasmids were overexpressed in PEDV-inoculated Vero-E6 cells to determine the effect of RPB2 and UPP1 on PEDV replication, and the viral replication level detected via IFA and Western blotting. The results showed that, compared with PEDV infection alone, after RPB2 overexpression, the expression levels of the PEDV N protein increased (Figure 6A), the PEDV-N protein-specific green fluorescence and the syncytia were increased (Figure 6B). As for UPP1, after overexpression, the expression levels of the PEDV N protein downregulated (Figure 6A), the PEDV-N protein-specific green fluorescence and the syncytia were also downregulated (Figure 6B).

### 3.7 Prediction of the interaction sites between PEDV N and the two host proteins

**Figure 7:**
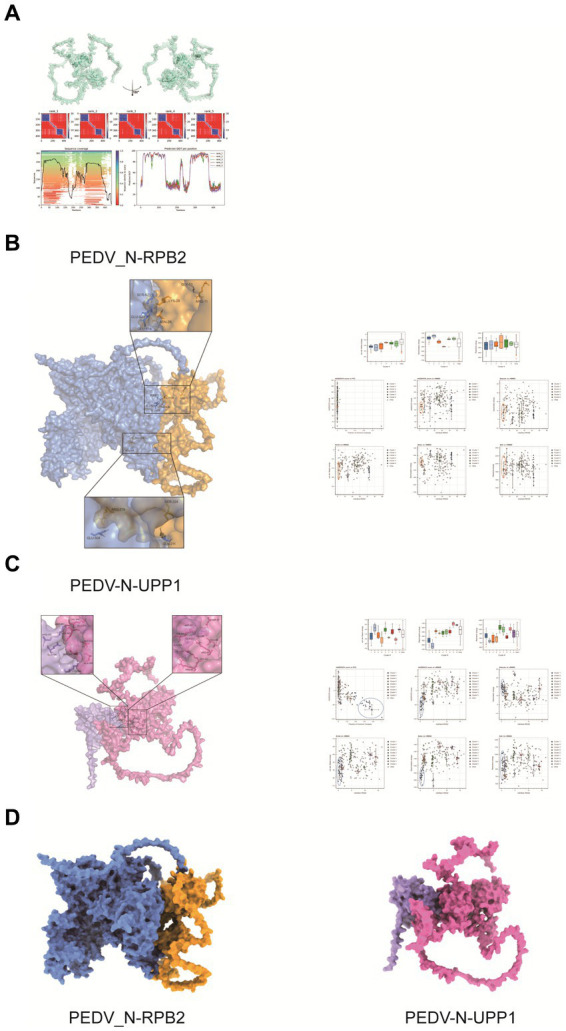
Predicted interaction sites of porcine epidemic diarrhea virus protein N (PEDV N) protein with host proteins RPB2 and UPP1. **(A)** N protein tertiary structure. **(B,C)** Protein interactions site prediction. In **B**, the blue part represents PEDV N protein, and the orange part represents RPB2. In **C**, the pink part represents PEDV N protein, and the purple part represents UPP1. **(D)** Optimal model prediction selection. In **D**, the blue part of PEDV_N-RPB2 represents PEDV N protein, and the orange part represents RPB2. The pink part of PEDV_N-UPP1 represents PEDV N protein, and the purple part represents UPP1.

Interaction sites between the PEDV N and host RPB2 and UPP1 proteins remain unclear. Hence, in pursuit of a comprehensive perception regarding the intricate interaction mechanisms exhibited between the PEDV N protein and host proteins RPB2 and UPP1, it is crucial to embark on advanced research. HADDOCK was used for model interaction prediction. The cluster was classified according to the affinity index, Van der Waals forces, proportion of contacting residues, restraints energy, and other parameters in the molecular docking of viral and host proteins. N protein tertiary structure was shown in Figure 7A. The results showed the optimal prediction models for the PEDV N and host RPB2 and UPP1 proteins were Cluster_4 and Cluster_1, respectively (Figures 7B,C). As claimed by the ultimate interaction model in HADDOCK, PDBePISA and PyMOL were carried out for interaction site selection. In the PDBePISA table, Structure refers to the amino acid residues and their corresponding positions, while HSDC represents the polar bond of the amino acid residue interaction, and ASA as well as BSA denote to the accessible surface area and the buried surface area separately, with ΔG corresponding to the folding free energy. At the interaction interface, both the ASA and BSA attain significant elevated score, indicating that the surface area exposed to the solvent and the hidden surface area were substantial. Consequently, the folding state of the protein was relatively stable and the folding free energy negative, which also indicates the flexibility and dynamics of the structure and corresponding region.

The predicted sites of amino acid interactions of PEDV N protein with host RPB2 and UPP1 were PEDV_N-RPB2: ARG-11 vs. GLY-53 and ARG-219 vs. GLU-504, PEDV-N-UPP1: ASP-27/ARG-60/GLU-68 vs. LYS-230 and ARG-63 vs. GLU-237 (Figures 7B,C). Figure 7D shows the conformational display of the 3D model of PEDV N protein interactions with host RPB2 and UPP1 proteins, providing a basis for studying interactions between the virus and host proteins.

## 4 Discussion

PED first broke out in the United Kingdom in 1971 and has become the primary cause of diarrheal diseases in pigs (22). PEDV N protein plays an important role in the process of virus infection. The PEDV N protein has been reported to play a role in recruiting the E3 ubiquitin ligase, COP1, and inhibiting COP1 self-ubiquitination and protein degradation, thus enhancing COP1 mediated p53 degradation and promoting viral replication (23). The PEDV N protein can degrade STAT1 by inhibiting ACE2 promoter activity and preventing its phosphorylation, thus inhibiting interferon-stimulated gene expression (14). Previous studies have explored how PEDV hijects PABPC1 and eIF4F proteins related to the host transcription translation system to promote viral proliferation, and promotes cyclization of viral mRNA carried by N protein, thus promoting viral transcription and promoting viral replication (13, 16). In this study, we explored the influence of PEDV N protein interaction with pyrimidine and purine metabolism pathway related proteins RPB2 and UPP1 on virus replication. LC-MS analysis and verification showed that RPB2 and UPP1 interact with PEDV N protein, and overexpression of RPB2 can promote PEDV replication, while overexpression of UPP1 can inhibit PEDV replication.

Eukaryotic RNA polymerase II comprises 12 subunits (RPB1-RPB12), of which RPB1 and RPB2 are the main subunits that constitute its catalytic center. They also play an important role in eukaryotic transcription (24). RPB affects gene expression levels through transcription initiation, transcription rate, transcription termination, and regulatory complex assembly. Viruses interact with factors associated with the host cell transcription system to regulate the extent of infection, further expansion, or suppression (17, 25). Herpes simplex virus (HSV) infection is known to promote complex formation of the RPB1 protein (26). BET inhibitors were reported to promote the recruitment of bromodomain-containing protein 4 and the CDK9/RPB1 complex to the HSV gene promoter, thus enhancing viral replication (27). The viral RNA-dependent RNA polymerase (FluPol) of the influenza A virus (IAV) binds to the regulatory CTD domain of RPB1 and interacts with RPB4 to initiate host transcription and secondary transcription of RPB4 (28). Nonstructural protein 2 of Chikungunya viruses (CHIKV) and Semliki Forest viruses (SFV) inhibits the IFN response by inducing the degradation of RPB1 (29, 30). In the purine and pyrimidine metabolism pathways enriched by host proteins that interacted with the PEDV N protein, as screened in this study, the RPB2 protein was present in both of them; thus, its influence on PEDV replication could be verified further. The PEDV N protein interacted with the host RPB2 protein, and overexpression of RPB2 was conducive to viral replication. It is speculated that the PEDV N protein may regulate the activity and stability of the RNA polymerase complex through interaction with RPB2 and improve its catalytic efficiency to promote viral self-replication. However, this hypothesis warrants further study.

UPP1 catalyzes the reversible phosphorylation of uridine (or 2′-deoxyuridine) to uracil and ribo-1-phosphate (or deoxyribo-1-phosphate) (18). It is mainly associated with immune and inflammatory responses, particularly T-cell activation (31). Studies have shown that berberine treatment inhibits pro-inflammatory and IRF8-IFN-γ signaling axis-related genes, including UPP1, *in vitro* and *in vivo* (32). In terms of energy metabolism, UPP1 can release uridine-derived ribose and promote central carbon metabolism, and its expression affects uridine utilization by cells (33). In the present study, we found that the PEDV N protein interacted with the host UPP1 protein, and UPP1 overexpression inhibited PEDV replication, which may be related to the regulation of host cell energy metabolism and the antiviral immune response by UPP1.

In summary, 144 host proteins that might interact with PEDV N proteins were screened using Co-IP and LC/MS-MS analyses. These host proteins were mainly concentrated in metabolic pathways, of which pyrimidine and urine metabolism were the most significant. In this study, two host proteins involved in pyrimidine and urine metabolism (RPB2 and UPP1) were verified, and the results showed that both proteins interacted with the PEDV N protein. Overexpression of RPB2 was found to promote PEDV replication, whereas overexpression of UPP1 inhibited PEDV replication. In addition, the predicted sites of amino acid interactions of PEDV N protein with host RPB2 and UPP1 were PEDV_N-RPB2: ARG-11 vs. GLY-53 and ARG-219 vs. GLU-504, PEDV-N-UPP1: ASP-27/ARG-60/GLU-68 vs. LYS-230 and ARG-63 vs. GLU-237. Overall, this study elucidated the interaction between two host proteins RPB2 and UPP1 related to nucleotide metabolism and PEDV N protein, which provided a theoretical basis for further exploring the pathogenesis and prevention of PEDV.

## Data availability statement

The datasets presented in this study can be found in online repositories. The names of the repository/repositories and accession number(s) can be found at: https://www.ebi.ac.uk/pride/archive/projects/PXD052564.

## Author contributions

YX: Conceptualization, Data curation, Validation, Writing – original draft. HY: Software, Writing – original draft, Conceptualization. QK: Formal analysis, Methodology, Software, Writing – original draft. XZ: Writing – review & editing, Conceptualization, Formal analysis. DX: Writing – review & editing, Investigation. LG: Writing – review & editing, Resources. LY: Writing – review & editing, Project administration, Supervision. BX: Funding acquisition, Visualization, Writing – review & editing.

## Footnotes

^1^ http://www.omicsbean.cn
